# Positive and Negative Leadership in Late Childhood: Similarities in Individual but Differences in Interpersonal Characteristics

**DOI:** 10.1007/s10964-023-01798-3

**Published:** 2023-06-12

**Authors:** Zhe Dong, Gijs Huitsing, René Veenstra

**Affiliations:** Department of Sociology, University of Groningen, Grote Rozenstraat 31, 9712 TG Groningen, The Netherlands

**Keywords:** leadership, popularity, bullying, defending, children, heterogeneity

## Abstract

Previous research has shown that leadership is associated not only with positive but also with negative characteristics and behaviors; knowledge of the similarities and differences between positive and negative leaders remains insufficient. This study aimed to examine (1) the existence of different subtypes of leaders and (2) to what extent these leaders differed on individual and interpersonal characteristics. The sample contained 9213 students in grades 3–6 (Dutch grades 5–8), from 392 classrooms in 98 schools (50.3% girls, *M*_age_ = 10.13 ± 1.23 years). Latent profile analysis identified three leader profiles and four non-leader profiles based on peer nominations received for leadership, popularity, and positive (defending) and negative (bullying) behavior: (1) positive leaders, (2) negative leaders, (3) non-popular leaders, (4) popular children, (5) bullies, (6) extreme bullies, and (7) modal children. Multinomial logistic regression showed similarities and differences between positive and negative leaders, as well as between each of these and the other five profiles. Positive leaders were more accepted and less rejected and had more friendships than negative leaders, but the differences in individual characteristics (self-esteem, self-control, and social goals) were less clear. This study demonstrated that 10–15% of the children were perceived as leaders, and that positive leadership became more prevalent in the higher grades. Nevertheless, negative leadership occurred also in the higher grades. Interventions aimed at turning negative leaders into positive leaders may work, because positive and negative leaders do not differ greatly in individual characteristics. Such interventions may improve the relationships of negative leaders with their classmates, which may be good for their likeability (but not at the expense of their popularity) as well as for the social atmosphere in the class as a whole.

## Introduction

Leaders are important for the social atmosphere and functioning of a classroom. Leadership has been argued to be a different construct from popularity, with no straightforward relation to it (Cillessen et al., 2014). Most definitions of leadership refer to coordination in steering the efforts of group members, the achievement of group goals, and social influence (Tackett et al., 2022). Leaders set and maintain social norms that function as guidelines for other children, as they give orders to classmates, make group decisions, and have power over others (Stavans & Diesendruck, 2021). Although leadership has been examined among adults, it has largely been neglected in research among children and adolescents (Tackett et al., 2022). Leaders are often stereotyped as prosocial and responsible children (Stavans & Diesendruck, 2021), but can also be bullies (Hawley, 2003). This underscores the urgency of distinguishing the positive and negative sides of leadership in childhood, and highlights ways of identifying leadership profiles through a person-centered approach (Tackett et al., 2022). This study aimed to examine whether there are different types of leaders in late childhood, and how these types can be explained. Latent profile analysis was used to explore distinct subtypes of children based on peer nominations for leadership, positive (defending) and negative (bullying) behavior, and their position in the social hierarchy (perceived popularity). Furthermore, the profiles were associated with individual and interpersonal characteristics to articulate key aspects of leadership. In this way, the current study introduces the concept of leadership in peer research in late childhood, and examines leadership in grades 3–6, enabling investigation of the development of leadership over the grades.

Positive and negative leaders are involved in group processes in different ways (Stavans & Diesendruck, 2021). Positive leaders have legitimate power and obtain social status by making decisions that are good for the group (Stavans & Diesendruck, 2021). Their focus is on “getting along” (Hawley, 2003; Hogan (1982)), and they use their skills for promoting relationships, fostering friendships, and prosocial behaviors, such as defending victims (Dijkstra et al., 2008). Well-liked leaders receive the most nominations for prosocial behavior, are high on perceived popularity, and hardly bully others (Andrews, 2020).While positive leaders use their social influence to provide concrete guidance to group members in order to facilitate prosocial behaviors, such as cooperation and defending (Andrews, 2020), other leaders may exert their influence to foster negative behaviors.

Negative leaders may dominate others and use aggressive behaviors to get what they want (Hawley, 1999; Peeters et al., 2010). Negative leaders combine aggressive behaviors and positive characteristics simultaneously (Hawley, 2003), and have a high social ranking, but achieve their social goals through intimidation and force (Stavans & Diesendruck, 2021). They compete with others and use the group for their own benefit. Their focus is on “getting ahead” (Hawley, 2003; Hogan (1982)). Negative leaders are likely to bully others, and their behaviors relate to demonstrating high social status and a strong desire for popularity (Lansford et al., 2009).

Although leadership has been linked to popularity as an antecedent or outcome (Farmer et al., 2003), it is unclear whether all popular children take up a leadership role. Popular children have been distinguished as prosocial popular leaders, antisocial popular leaders, prosocial popular non-leaders, and antisocial popular non-leaders (Andrews, 2020; Farmer et al., 2003). Popular leaders stand high in the hierarchy and obtain privileged resources. They may use coercive (aggressive, e.g., bullying) as well as prosocial (cooperation, e.g., defending) strategies to maintain their social position (Hawley, 1999). In other words, popular leaders are expected to be a heterogeneous group.

Given the existence of different types of leaders, it is also relevant to examine their differences. The Leadership Trait Theory (Zaccaro et al., 2004) has classified distal and proximal attributes of leaders. Distal attributes refer to individual characteristics (e.g., personality) that enable children to become leaders rather than followers (Judge et al., 2009). Relevant individual factors are self-esteem, self-control, and social goals. Self-esteem refers to people’s attitudes and self-perception (Rosenberg, 1965). Children with higher self-esteem have more confidence and motivation to become leaders (Yuan et al., 2020). Self-control reflects the ability to invest in long-term benefits rather than to be tempted by immediate rewards, and may be important for both positive and negative leaders (Gagne, 2017). Whereas self-esteem and self-control may be similarly high in positive and negative leaders, these types of leaders may differ in social goals. Social goals reflect a general orientation toward relationships, and can be divided into social development goals (referring to an improvement of friendship skills in order to realize high-quality relationships), demonstration-avoidance goals (referring to the avoidance of embarrassment and others’ negative judgments), and demonstration-approach goals (referring to obtaining favorable judgments from others and social prestige; Rudolph et al., 2011). Social development goals and demonstration-avoidance goals may characterize positive leaders, whereas demonstration-approach goals may characterize negative leaders.

Proximal attributes are about interpersonal skills, including the style leaders perform to get along with followers, and social appraisal, which relies on others’ subjective evaluations and indicates leadership effectiveness (Judge et al., 2009). Proximal attributes indicate leaders’ ability to understand the feelings, thoughts, and behaviors of others. In expressing leadership, leaders show the ability to deal with group dynamics, understand and earn trust from group members, and allocate resources to satisfy classmates’ expectations (Hawley, 1999). Because positive leaders focus on getting along with others, it is likely that they score positively on interpersonal factors. The focus of negative leaders on getting ahead may relate to more negative interpersonal characteristics, such as a lower number of friends, less peer acceptance, and more peer rejection. In addition, earlier research found that boys were more likely to be leaders (Eva et al., 2021), and that leadership was more prevalent among students in the higher grades (Dhuey & Lipscomb, 2008).

## Current Study

The similarities and differences between positive and negative leaders are unknown. This study aimed to explore the presence of distinct leadership profiles in late childhood, and whether children’s individual and interpersonal characteristics were related to positive and negative leadership. First, the profiles were based on children’s scores for leadership, perceived popularity, and prosocial (defending) and antisocial (bullying) behavior. Using a person-centered approach, two subtypes of leaders were expected. Positive leaders would display leadership, above-average popularity, and positive (defending) behavior, and would not be involved in bullying; negative leaders would be similar to positive leaders in terms of leadership and popularity, but differ in bullying and defending (Hypothesis 1a). It was expected that negative leaders would have higher involvement in negative behavior and lower involvement in positive behavior. Gender and grade differences in leadership were controlled for. It was hypothesized that boys would be overrepresented among both positive and negative leaders (Hypothesis 1b). Furthermore, it was expected that children in the higher grades would more often be perceived as positive and negative leaders than children in the lower grades (Hypothesis 1c). Next, the individual and interpersonal attributes of these profiles were examined. Relevant measures at the individual level were self-esteem, self-control, and social goals. At the interpersonal level, number of friends, social acceptance and rejection, and self-reported victimization were included. At the individual level, it was hypothesized that positive and negative leaders’ social goals would align with their social position of. Positive leaders would have higher social development goals, aiming for good friendships, and higher demonstration-avoidance goals, referring to the avoidance of standing out negatively, and higher levels of self-esteem and self-control. Negative leaders would have higher demonstration-approach goals, referring to being visible and dominant, and also higher levels of self-esteem and self-control than non-leaders (Hypothesis 2). In addition, at the interpersonal level, it was hypothesized that positive leaders would have more favorable interpersonal characteristics than negative leaders, higher levels of friendships and acceptance, and lower levels of rejection and self-reported victimization (Hypothesis 3).

## Methods

### Procedure

Data used in this study stem from the KiVa anti-bullying program in the Netherlands (Huitsing et al., 2020; Veenstra et al., 2020). The data were collected in May and October 2012, before and at the start of the intervention (Huitsing et al., 2020).

Before the pre-assessment in May 2012, a KiVa information guide and consent forms were sent to parents, to allow their children to participate. Passive parental and student consent was obtained: parents could opt their children out of participation, and students themselves could also opt out of the assessment at any time. The response rate at each of the five waves exceeded 95%. Students completed the questionnaires online in the school computer labs, during regular school hours, under teachers’ guidance, and they could ask teachers for help when necessary. Students who missed the scheduled day of data collection could participate another day within a month. The questions of each scale were presented in random order, to avoid the possibility that the order might systematically affect the results.

For the peer nomination questions, a list of the names of classmates was provided for the students to choose from: they were asked to nominate an unlimited number of classmates for all peer nomination questions. Students could nominate the same peer for more than one question, and were allowed to nominate absent peers. To take differences in classroom size into account, the number of peer nominations each child received from participating classmates was converted into a proportion score.

### Sample

The sample comprised a total of 9213 students (*M*_age_ = 10.13, SD = 1.23; 50.3% girls) from 392 classrooms (mean classroom size was 23.50, *SD* = 6.11) in 98 Dutch schools, from grades 3–6 (Dutch grades 5–8).

### Measures

Most measures were collected at T2 (October 2012). Some measures were collected at T1 (May 2012) to reduce the shared method variance between the measures for leadership and popularity (both assessed at T2) and the interpersonal characteristics (all assessed at T1).

#### Leadership (T2)

To ensure that the children understood the meaning of leadership, the concept was explained (“Do you know what a good leader is? A leader is someone who often determines what needs to be done. Such as the captain of a team or a coach. They often say what others have to do”). The proportion score for the number of nominations children received from their classmates on the question “Are there children in your class who are leaders? Which classmates are good leaders?” was used to indicate leadership.

#### Popularity (T2)

To ensure that the children understood the meaning of popularity, this concept was explained (“Popular children are children that others want to hang out with. Popular children are cool”). The proportion score for the number of nominations children received from their classmates on the question “Which classmates are popular?” was used to indicate popularity.

#### Peer-reported bullying and defending (T2)

To measure bullying and defending, children were first asked whether they were being victimized on any of the 11 self-reported Olweus’ (1996) bully/victim items (concerning several forms of victimization; see Kaufman et al., 2020). After watching an instructional video, in which the definition of bullying was explained (i.e., repeatedly harassing another child, with the victim having problems defending him or herself), participants responded to the bully/victim questionnaire. If they indicated that they had been victimized at least once on any item, they were asked whether they were victimized by classmates, other students from the school, or others outside the school. If children reported being victimized by classmates, they were asked “Who starts when you are victimized?” to indicate their bullies. Defending was explained, too (“Defending is helping, supporting, or comforting victimized students”), and victimized children were asked “Which classmates defend you when you are victimized?” to indicate their defenders. Proportion scores for the numbers of nominations children received from their classmates were calculated for bullying and defending.

#### Other peer-reported variables (T1)

The proportion scores for the numbers of nominations children received from their classmates on the questions (1) Which classmates do you like?; (2) Which classmates do you dislike?; and (3) Which classmates are your best friends? was used to indicate liking, disliking, and number of friends.

#### Self-esteem (T2)

Self-esteem was measured using a five-item scale derived from the Rosenberg self-esteem scale (Huitsing et al., 2019; Rosenberg, 1965); only positively formulated items were used for this age group (e.g., I feel that I am a person of worth, at least on an equal plane with others). This is a five-point Likert-type scale (0 = never, 4 = always), with Cronbach’s alpha = 0.84.

#### Self-control (T2)

Self-control was measured using eight items from the Temperament in Middle Childhood Questionnaire (CMPQ) (Simonds & Rothbart, 2004). Students responded on a five-point scale (1 = not at all to 5 = always). Three items were reverse coded before creating the scale (I talk before thinking; I interrupt people when they’re talking; I get into trouble because I do things without thinking first), with Cronbach’s alpha = 0.69.

#### Social goals (T2)

Social goals were measured using the shortened version of the Social Achievement Goals Questionnaire (Rudolph et al., 2011). The short version contains nine items, which students answered on a five-point scale (1 = not at all to 5 = very often). The questionnaire contains three subscales: social development goals (e.g., I try to figure out what makes a good friend), demonstration-approach goals (e.g., It is important to me to have “cool” friends), and demonstration-avoidance goals (e.g., One of my main goals is to make sure other children don’t say anything bad about me). The Cronbach’s alphas for these three subscales were 0.80, 0.77, and 0.78, respectively.

#### Self-reported victimization (T1)

Self-reported victimization was measured using the Revised Olweus Bully/Victim questionnaire (Olweus, 1996). Participants responded to a global question (“How often have you been bullied during the past couple of months?”) and seven specific items concerning physical, verbal (two items), relational (two items), material (i.e., taking or breaking others’ property), and cyberbullying. This is a five-point scale (0 = not at all, 1 = once or twice, 2 = two or three times a month, 3 = about once a week, 4 = several times per week), with Cronbach’s alpha = 0.87.

#### Control variables

Gender (1 = boys; 0 = girls) and grade were used as control variables. Three ordered dummies were used for grade: being in grade 4 or higher (children in grade 3 = 0, children in grade 4 or higher = 1), being in grade 5 or higher (children in grades 3 and 4 = 0, children in grades 5 and 6 = 1), and being in grade 6 (children in grade 5 or lower = 0, children in grade 6 = 1).

### Analytical Strategy

The analyses were divided into two steps. First, latent profile analysis (LPA) was applied in M*plus* 8.3 (Muthén & Muthén, 2012) to investigate the heterogeneity of leadership types. The profiles examined in this study were based on the *z*-standardized scores of peer-reported leadership, defending, bullying, and popularity. Two- to eight-profile models were compared in order to evaluate the optimal number of profiles (Nylund et al., 2007). Following previous research (Gabriel et al., 2015), the model fit was estimated using the following seven indices: log-likelihood (LL), Akaike information criterion (AIC), Bayesian information criterion (BIC), sample-size-adjusted BIC (SSA-BIC, recommended by Tofighi & Enders, 2007), Lo-Mendell-Rubin likelihood ratio test (LMR-LRT; Lo et al., 2001), bootstrap likelihood ratio tests (BLRT), and entropy. The best-fitting model was determined as follows: (1) LL, AIC, BIC, and SSA-BIC should be lower than other profile solutions; (2) entropy should be higher than other profile solutions (values range from 0 to 1; values > 0.8 indicate that the accuracy of model classification exceeds 90%); (3) LMR and BLRT should be significant (*p* < 0.05); (4) BIC requires at least 50 children per profile to be >90% accurate (recommended by Yang, 2006); (5) post hoc tests were conducted to examine whether indicators’ mean difference between each pair showed significance; if most mean differences did not show significance, the best *n*-1 type would be selected; (6) the theoretical meaning of each profile was considered in the selection of the best solution.

Second, the means of individual and interpersonal attributes were compared for the different leadership profiles using univariate ANOVA, including post hoc Scheffé tests in SPSS. Third, the multinomial logistic model (MNLM) was used to examine the multivariate relation between individual and interpersonal characteristics and the different leadership profiles. The MNLM can examine the effects of independent variables on a nominal dependent variable, such as leadership profiles. With seven outcomes, the MLNM is roughly equivalent to running 21 binary logistic regressions comparing outcomes (e.g., comparing profile 1 to profiles 2–7, comparing profile 2 to profiles 3–7, etc.). In the MNLM, all of the logits are estimated simultaneously, which emphasizes the logical relations among the parameters and uses the data more efficiently (Long, 1997). Marginal effects were calculated to interpret the outcomes of the MNLM (Borooah, 2002; Liao, 1994). For a dummy variable, the marginal effect is the effect of being in Category 1 rather than in Category 0 (for more details, see Veenstra et al., 2005). For continuous variables, the marginal effect is the effect of a one-point increase in an attribute on an outcome. The marginal effects sum up to zero per variable.

There were no missing data for popularity, leadership, bullying, liking, disliking, friendship, and defending, because all children received nominations from their classmates. Missing data were low for self-esteem (*N* = 60, 0.7%), self-control (*N* = 112, 1.2%), social goals (*N* = 51, 0.6%), and self-reported victimization (*N* = 616, 6.7%). STATA used listwise deletion for attributes and kept 8,503 children in the multinomial logistic models (MNLM).

## Results

### Descriptive Statistics

Table 1 provides the descriptive statistics and correlations of the variables. Boys were seen as leaders slightly more often than girls (*M*_boys_ = 0.15, *M*_girls_ = 0.14, *t*_(9213)_ = 17.89, *p* < 0.01). Leadership correlated positively with popularity, defending, and bullying. It also correlated positively with peer acceptance and friendships, and negatively with peer rejection and victimization.

**Table 1:** Descriptive Statistics and Correlations for Study Variables for Girls (above the diagonal) and Boys (below the diagonal)

Variables	M	SD	1	2	3	4	5	6	7	8	9	10	11	12	13	14	15	16
1. Leadership	0.15	0.13	1	0.51	0.42	0.11	0.07	0.03	−0.00	−0.00	−0.02	0.37	0.36	−0.18	−0.05	−0.06	−0.08	0.03
2. Popularity	0.14	0.17	0.57	1	0.30	0.18	0.01	−0.05	−0.08	−0.03	0.06	0.30	0.31	−0.16	−0.08	0.09	0.15	0.22
3. Defending	0.10	0.08	0.40	0.27	1	0.01	0.01	0.00	0.03	−0.06	−0.01	0.39	0.31	−0.17	0.00	−0.12	−0.17	−0.10
4. Bullying	0.03	0.06	0.13	0.24	0.05	1	−0.05	−0.12	0.02	−0.04	0.08	0.00	−0.11	0.31	0.18	−0.08	−0.09	−0.05
5. Self-esteem	2.92	0.88	0.07	0.07	−0.01	−0.07	1	0.34	0.04	0.10	0.02	0.02	0.05	−0.10	−0.15	0.05	0.03	0.01
6. Self-control	3.86	0.64	0.02	−0.09	0.02	−0.20	0.38	1	0.01	0.09	−0.15	0.03	0.07	−0.13	−0.16	−0.00	−0.04	−0.04
7. Social development goals	3.55	1.19	0.03	−0.08	0.04	−0.00	0.07	0.08	1	0.22	0.23	−0.01	−0.05	0.08	0.15	−0.10	−0.10	−0.10
8. Demonstration-avoidance goals	3.49	1.33	−0.03	−0.05	−0.07	−0.08	0.15	0.12	0.25	1	0.15	−0.07	0.00	−0.01	0.03	0.17	0.14	0.09
9. Demonstration-approach goals	2.49	1.12	0.06	0.10	0.03	0.14	0.07	−0.11	0.27	0.17	1	−0.02	−0.08	0.11	0.13	−0.07	−0.05	−0.02
10. Number of friends	0.25	0.14	0.36	0.26	0.36	−0.07	0.02	0.05	0.04	−0.07	0.04	1	0.66	−0.35	−0.10	−0.20	−0.20	−0.11
11. Social acceptance	0.42	0.18	0.31	0.28	0.26	−0.19	0.07	0.10	−0.00	−0.01	−0.05	0.67	1	−0.53	−0.22	0.07	0.08	0.11
12. Social rejection	0.15	0.15	−0.19	−0.16	−0.16	0.37	−0.09	−0.16	0.01	−0.03	0.09	−0.42	−0.62	1	0.35	−0.12	−0.13	−0.10
13. Self-reported victimization	1.65	0.78	−0.10	−0.15	0.00	0.12	−0.15	−0.14	0.12	0.04	0.10	−0.12	−0.21	0.30	1	−0.11	−0.13	−0.12
14. Being in grade 4 or higher	0.76	0.43	−0.08	0.13	−0.19	−0.11	0.12	0.01	−0.12	0.17	−0.12	−0.21	0.03	−0.10	−0.17	1	0.55	0.32
15. Being in grade 5 or higher	0.50	0.50	−0.07	0.16	−0.17	−0.12	0.10	−0.02	−0.13	0.15	−0.12	−0.20	0.09	−0.13	−0.17	0.59	1	0.58
16. Being in grade 6	0.25	0.43	0.03	0.19	−0.09	−0.07	0.07	−0.02	−0.08	0.11	−0.07	−0.14	0.08	−0.09	−0.11	0.33	0.56	1

All correlations ≥ |0.06| are significant at <0.001

### Identifying Types of Children with LPA

Table 2 presents the fit statistics for the two- to eight-profile solutions. The seven-profile solution was selected. The seven-profile solution had lower LL, AIC, BIC, and SSA-BIC than the two- to six-profile solutions. In addition, the seven-profile solution had reasonable sample sizes for each profile (163-6222) and an acceptable entropy value of 0.905. More than 90% of children were classified accurately, and post hoc tests showed significant differences in most comparisons. The eight-profile solution was rejected because it failed in the LMR value.

**Table 2:** Fit Statistics for Profile Structures

No. of profiles	LL	FP	AIC	BIC	SSA-BIC	LMR (*p*)	BLRT (*p*)	Entropy
2	−48786.7	13	97597.5	97690.1	97648.8	<0.0001	<0.0001	0.928
3	−47122.1	18	94280.2	94408.5	94351.3	<0.0001	<0.0001	0.942
4	−45926.7	23	91899.3	92063.3	91990.2	<0.0001	<0.0001	0.921
5	−47019.5	28	90630.9	90830.5	90741.5	<0.0001	<0.0001	0.902
6	−44690.7	33	89447.5	89682.7	89577.8	0.1880	<0.0001	0.896
**7**	−**44133.9**	**38**	**88343.8**	**88614.7**	**88493.4**	**0.0002**	**<0.0001**	**0.903**
8	−43687.3	43	87460.5	87767.0	87630.4	0.6679	<0.0001	0.907

The selected solution is in bold. *LL* log-likelihood, *FP* Number of free parameters, *AIC* Akaike information criterion, *BIC* Bayesian information criterion, *SSA-BIC* sample-size-adjusted BIC, *LMRT* Lo, Mendell, and Rubin (2001) likelihood ratio test; *BLRT* bootstrap likelihood ratio tests.

**Fig. 1:**
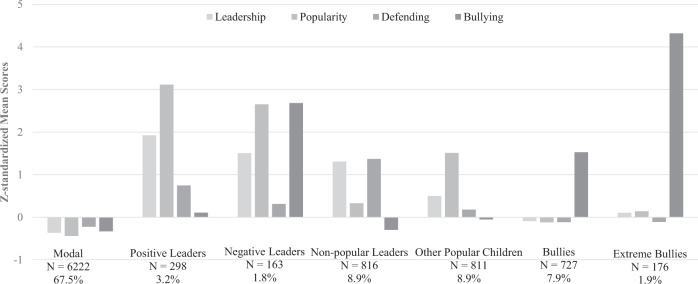
Profiles of Child Types Based on Peer Reports of the Seven-type Solution (*z*-standardized mean scores)

The seven profiles were labeled according to the estimated *z*-standardized mean indicator variables (see Table 3 and Fig. 1). The largest profile was labeled as modal (*N* = 6222); this comprised children characterized by below-average levels of leadership, popularity, defending, and bullying. The second and third profiles consisted of positive and negative leaders, who were both high on leadership and popularity but differed in defending and bullying. *Positive leaders* (3.2%, *N* = 298, 58.1% boys) were characterized by high levels of defending and low levels of bullying. In contrast, *negative leaders* (1.8%, *N* = 163, 82.5% boys) were characterized by medium levels of defending and high levels of bullying. Out of the 392 classrooms, 109 had only positive leaders, 62 had only negative leaders, 47 had both positive and negative leaders, and 174 had no positive or negative leaders. An additional profile of *non-popular leaders* (8.9%, *N* = 816, 38.7% boys) was also found. This profile had above-average leadership, the highest level of defending, and relatively low levels of popularity and bullying.

**Table 3:** Descriptive Information per Latent Profile

Type	Number	% of sample	% of boys	Leadership	Popularity	Defending	Bullying
1. Modal	6222	67.5	45.0	−0.365	−0.441	−0.224	−0.332
2. Positive Leaders	298	3.2	58.1	1.922	3.116	0.747	0.106
3. Negative Leaders	163	1.8	82.5	1.503	2.651	0.313	2.682
4. Non-popular leaders	816	8.9	38.7	1.308	0.326	1.368	−0.298
5. Other Popular Children	811	8.8	57.6	0.500	1.509	0.179	−0.053
6. Bullies	727	7.9	73.2	−0.092	−0.l20	−0.115	1.524
7. Extreme Bullies	176	1.9	84.1	0.105	0.139	−0.111	4.317

The other profiles that were found consisted of *other popular children* (8.8%, *N* = 811, 57.6% boys), who had high levels of popularity, moderate levels of leadership and defending, and low levels of bullying; and *pure bullies* (7.9%, *N* = 727, 73.2% boys) and *extreme bullies* (1.9%, *N* = 176, 84.1% boys), who scored high on bullying, and (below) average on leadership, popularity, and defending.

To examine the generalizability of the leader profiles, the latent profile analyses were replicated using the T1 data of KiVa NL. Largely the same children participated in T1 and T2, but the classmates differed because most Dutch schools have mixed-grade classrooms (Rambaran et al., 2019). Appendix 1 shows the fit statistics for the profile structures and the descriptive information per latent profile. Again, three profiles of leaders and four profiles of non-leaders were found.

### Univariate Analysis

Table 4 presents the attributes of the profiles and the post hoc Scheffé tests for differences between the profiles. Positive leaders, negative leaders, and other popular children were more often in the higher grades; non-popular leaders, bullies, and extreme bullies were more often in the lower grades.

**Table 4:** Descriptive Information for Attributes

Attributes	Modal	Positive Leaders	Negative Leaders	Non-popular Leaders	Other Popular Children	Bullies	Extreme Bullies	Cluster differences
Grade 3	63.9%	0.8%	0.5%	14.9%	3.5%	12.8%	3.6%	
Grade 4	69.0%	1.3%	1.8%	10.8%	6.1%	8.3%	2.6%	
Grade 5	74.1%	2.5%	2.0%	5.0%	9.6%	6.0%	0.8%	
Grade 6	62.9%	8.3%	2.6%	5.0%	15.9%	4.6%	0.7%	
Gender (1 boys and 0 girls)	0.45^ab^	0.58^c^	0.83^d^	0.39^a^	0.58^bc^	0.73^d^	0.84^d^	*F* = 77.07^**^
*Individual level*								
Self-esteem	2.92^bc^	3.03^c^	3.14^c^	2.94^bc^	3.02^c^	2.79^ab^	2.64^a^	*F* = 10.20^**^
Self-control	3.91^d^	3.74^bc^	3.55^a^	3.95^d^	3.80^cd^	3.61^ab^	3.47^a^	*F* = 47.68^**^
Social development goals	3.57^ab^	3.39^ab^	3.36^ab^	3.60^b^	3.28^a^	3.66^b^	3.56^ab^	*F* = 10.09^**^
Demonstration-avoidance goals	3.54^a^	3.47^a^	3.24^a^	3.43^a^	3.42^a^	3.28^a^	3.39^a^	*F* = 6.21^**^
Demonstration-approach goals	2.41^a^	2.64^ab^	3.00^c^	2.40^a^	2.62^ab^	2.81^bc^	3.06^c^	*F* = 32.12^**^
*Interpersonal level*								
Number of friends	0.22^a^	0.34^c^	0.27^b^	0.36^c^	0.30^c^	0.22^a^	0.21^a^	*F* = 179.88^**^
Liking (social acceptance)	0.40^c^	0.57^e^	0.42^c^	0.55^de^	0.51^d^	0.35^b^	0.29^a^	*F* = 188.54^**^
Disliking (social rejection)	0.15^b^	0.09^a^	0.23^c^	0.08^a^	0.10^a^	0.27^d^	0.37^e^	*F* = 237.63^**^
Self-reported victimization	1.64^b^	1.42^a^	1.48^ab^	1.59^ab^	1.45^ab^	1.96^c^	2.10^c^	*F* = 43.06^**^

All values were estimated from ANOVA Means in the same row that do not share superscripts differ at *p* < 0.05 in the Scheffé test

Positive leaders differed in several aspects from negative leaders. Boys were overrepresented in both groups, but more so among negative leaders. At the individual level, positive leaders had higher self-control and fewer demonstration-approach goals than negative leaders. Negative and positive leaders had the highest self-esteem and differed significantly from (extreme) bullies. At the interpersonal level, positive leaders were more accepted, less rejected, and had more friendships than negative leaders. The third group of leaders was the non-popular leaders. Girls were overrepresented in this group. At the individual level, non-popular leaders had the highest self-control and the lowest demonstration-approach goals. Non-popular leaders also had higher social development goals than negative and positive leaders. At the interpersonal level, non-popular leaders resembled positive leaders.

Negative leaders and (extreme) bullies differed in interpersonal attributes, but not in individual attributes, except for self-esteem, which was lower among the (extreme) bullies. Compared with (extreme) bullies, negative leaders had more friends and were more often liked, less often disliked, and less often victimized.

### Multinomial Logistic Regression

Table 5 presents the marginal effects of the MNLM. The rows show the effects of the attributes on the likelihood of being in one of the seven profiles, with the effects of all other attributes controlled for. The sum of each row equals zero.

**Table 5:** Multinomial Logistic Regression on the Seven Types of Children: Marginal Effects (and Standard Errors) of Gender and the Individual and Interpersonal Attributes

	Modal	Positive Leaders	Negative Leaders	Non-popular Leaders	Other Popular Children	Bullies	Extreme Bullies
	(67.54%)	(3.24%)	(1.77%)	(8.86%)	(8.80%)	(7.89%)	(1.91%)
Grades 4/5/6	−4.36 (1.52)^**^	2.36 (0.93)^*^	2.47 (0.59)^**^	−3.01 (0.74)^**^	5.07 (1.18)^**^	−2.31 (0.72)^**^	−0.21 (0.34)
Grades 5/6	2.79 (0.14)	2.37 (0.70)^**^	0.40 (0.38)	−6.22 (0.91)^**^	3.75 (0.90)^**^	−1.58 (0.82)	−1.51 (0.49)^**^
Grade 6	−7.20 (1.42)^**^	3.75 (0.49)^**^	0.58 (0.35)	−0.06 (1.02)	4.24 (0.72)^**^	−1.36 (0.93)	0.06 (0.60)
Gender (1 boys and 0 girls)	−8.64 (1.00)^**^	0.65 (0.38)	1.51 (0.37)^**^	−2.55 (0.61)^**^	2.12 (0.62)^**^	4.97 (0.64)^**^	1.94 (0.40)^**^
*Individual level*							
Self-esteem	−1.07 (0.52)^*^	0.28 (0.22)	0.33 (0.16)^*^	0.13 (0.33)	0.53 (0.36)	−0.03 (0.27)	−0.18 (0.13)
Self-control	3.60 (0.52)^**^	−0.58 (0.22)^**^	−0.52 (0.15)^**^	0.20 (0.33)^*^	−0.79 (0.34)^**^	−1.46 (0.28)^**^	−0.45 (0.14)^**^
Social development goals	1.60 (0.51)^**^	−0.07 (0.19)^**^	−0.12 (0.14)	0.04 (0.31)	−1.49 (0.31)^**^	0.30 (0.30)	−0.25 (0.15)
Demonstration-avoidance goals	2.08 (0.51)^**^	−0.58 (0.21)^**^	−0.37 (0.16)	0.53 (0.30)	−1.11 (0.33)^**^	−0.72 (0.29)^*^	0.17 (0.16)
Demonstration-approach goals	−3.53 (0.51)^**^	0.79 (0.21)^**^	0.59 (0.15) ^**^	−0.71 (0.32)^*^	1.97 (0.32)^**^	0.65 (0.28)^*^	0.25 (0.14)
*Interpersonal level*							
Number of friends	−9.74 (0.67)^**^	2.01 (0.25)^**^	0.97 (0.21)^**^	2.60 (0.36)^**^	3.30 (0.41)^**^	0.13 (0.42)	0.73 (0.23)^**^
Liking (social acceptance)	−4.95 (0.74)^**^	0.97 (0.27)^**^	0.06 (0.23)	2.87 (0.44)^**^	0.78 (0.44)	0.63 (0.47)	−0.36 (0.27)
Disliking (social rejection)	−2.87 (0.69)^**^	0.39 (0.29)	1.22 (0.18)^**^	−2.94 (0.52)^**^	−1.50 (0.48)^**^	4.17 (0.31)^**^	1.53 (0.17)^**^
Self-reported victimization	0.73 (0.54)	−0.36 (0.26)	−0.56 (0.19) ^**^	0.14 (0.33)	−0.73 (0.40)	0.69 (0.25)^**^	0.08 (0.12)

*N* = 8503 ^*^p < 0.05; ^**^p < 0.01

As shown in Table 5, and in line with the *grade hypothesis*, particularly positive leaders were more often in the higher grades, whereas non-popular leaders were more likely to be in the lowest grade. In line with the *gender hypothesis*, boys were more likely to be positive and negative leaders, other popular children, or (extreme) bullies. Girls were more likely to be in the modal profile or to be among the non-popular leaders.

At the individual level, when children scored one SD above the mean on self-esteem and demonstration-approach goals and one SD below the mean on self-control, social development goals, and demonstration-avoidance goals, their chances of being positive leaders increased from 3.24% (the group size of positive leaders) to 5.42% (an increase of 71%), and their chances of being negative leaders increased from 1.77% to 3.70% (an increase of 109%). These findings are contrary to the individual-level hypothesis: positive and negative leaders do not differ substantively in self-esteem, self-control, and social goals.

At the interpersonal level, scoring one SD above the mean on both number of friends and liking and one SD below the mean on both disliking and victimization increased the chances of being a positive leader from 3.24% to 6.19% (an increase of 94%), whereas the chances of being a negative leader increased slightly from 1.77% to 2.14% (an increase of 21%). In line with the interpersonal-level hypothesis, leaders with more friendships and acceptance, less rejection, and less self-reported victimization were more likely to be positive than negative leaders.

## Discussion

Researchers have long ignored the styles and traits of popular leaders in late childhood. Although they are often associated with prosocial behaviors and positive characteristics, there are also negative leaders. The aim of this study was to examine the existence of subtypes of leaders and explore similarities and differences in their individual and interpersonal characteristics. Latent profile analysis indicated that three leader and four non-leader profiles could be distinguished based on nominations received for leadership, positive (defending) and negative (bullying) behavior, and popularity. Positive and negative leaders were quite similar in individual characteristics, but differed in interpersonal factors: positive leaders had more friends and were more accepted, less rejected, and less victimized than negative leaders. These findings represent a first step in examining the attributes of leadership in late childhood.

Three types of leaders were found, demonstrating heterogeneity in leadership. In total, 13.9% of the children belonged to one of the leader profiles. Positive leaders received the most nominations for leadership and popularity as well as for defending, but they were hardly nominated for bullying, which is in line with the profile of well-liked leaders (Andrews, 2020). *Negative leaders* combined high levels of leadership and popularity with high levels of bullying and relatively low levels of defending. Negative leaders reflected more of the characteristics of bi-strategic controllers, also labeled as “well-adapted Machiavellian” (Hawley, 2003). The third group was the non-popular leaders, who scored the highest on defending and were also high on leadership, but had relatively low levels of popularity and scored very low on bullying. Thus, popular leaders could be divided into positive and negative leaders, whereas non-popular leaders formed a group of children that stood out as defenders.

In addition to the leader profiles, four non-leaders profiles were found. *Other popular children* were popular without being involved in defending or bullying. These children might obtain their popularity through other means: for example, by being athletic or physically attractive (Dijkstra et al., 2008). In addition, two types of pure bullies were found. They differed in how much they bullied. Finally, about two-thirds of the students in this sample were in the modal group and received below-average nominations for leadership, positive and negative behavior, and social status.

### Explaining Leadership Types

In line with the Leadership Trait Theory, this study examined individual and interpersonal characteristics of positive and negative leaders (Zaccaro et al., 2004). In the univariate analyses, negative leaders had higher levels of demonstration-approach goals than positive (or non-popular) leaders; thus, their goal was to be visible and stand out. However, this effect disappeared in the multinomial logistic regression model. In addition, the three groups of leaders had the highest levels of self-esteem. In line with the hypothesis, self-esteem effectively differentiates leaders from non-leaders. Children who have high self-esteem are better able to deal with challenges and problems in group processes and peer interactions (Liu et al., 2019). Surprisingly, children with higher self-control were less likely to be positive or negative leaders.

In line with the interpersonal-level hypothesis, positive leaders had a more favorable social position than negative leaders. Positive (and also non-popular) leaders had more friends, were more socially accepted, and were less socially rejected by peers than negative leaders. Thus, following Leadership Trait Theory, interpersonal-level factors differentiate positive and negative leadership styles.

In line with the grade hypothesis, most leaders in grades 3-4 were non-popular, whereas in grade 5, and particularly grade 6, the leaders were more often seen as positive or negative leaders. In the higher grades, the likelihood of finding leaders who are not popular declines. The establishment of a clear hierarchy at the end of primary school might explain this. Popular children set the norms, and positive leaders use their dominant position for getting along with others, which puts attention on the group and common goals, and may lead to the defending of victims; negative leaders use their position for getting ahead by commanding attention in a self-serving way and pursuing self-orientation goals, including through the bullying of classmates.

Boys were overrepresented among positive leaders and, particularly, among negative leaders, whereas girls were overrepresented among non-popular leaders; this aligns with the gender hypothesis. A possible explanation is that girls’ leadership patterns may focus on taking care of others and being kind, whereas boys’ leadership patterns may focus on taking charge and competition (Eva et al., 2021). As such, girl leaders are less likely to stand out by being dominant, which is often associated with popularity (Hawley, 2003). Non-popular leaders resembled positive leaders in terms of interpersonal characteristics. As well as having high levels of self-control and social development goals, the findings suggest that these (primarily female) leaders were likable and focused on harmonious relationships.

The latent profile analysis further generated two subtypes of pure bullies: bullies and extreme bullies. Like negative leaders, they had low levels of self-control; this is in line with previous studies (Moon & Alarid, 2015). In addition, these three profiles also had relatively high levels of demonstration-approach goals. The pure bullies were rarely nominated as friends, but scored surprisingly high on social-development goals. This suggests that they were willing to become better in friendships. Both profiles of bullies showed high levels of self-reported victimization, and may be seen as bully-victims (Veenstra et al., 2005).

### Strengths, Limitations, and Directions for Further Research

This study has several strengths, including a large sample, the combination of information on leadership with other status measures (popularity) and positive and negative behavior, and the examination of the attributes of leadership types. About 10–15% of the children were perceived as leaders, but the findings showed that this percentage was divided over three profiles. For that reason, if other researchers wish to replicate or extend our findings, they will need a large sample, too, because classrooms generally contain few leaders. This study has already replicated the finding of the seven profiles through a latent profile analysis in another school year, in which the composition of the classrooms differed largely, and as such the peer nominations were given by other classmates.

Notwithstanding the contributions made by this study, the results must be interpreted with the following limitations in mind. First, the findings were cross-sectional, precluding conclusions about the bidirectional associations between leadership types and attributes. Perhaps individual and interpersonal characteristics function as instigators of leadership behavior, but they may also be the result of a leadership position. Further research might test the bidirectional relations between leadership and these characteristics, as well as how leadership profiles change over time. Second, the peer nomination question about leadership focused on good leaders. This focus fits with the goal of the KiVa intervention to prevent bullying, improve the school atmosphere, and make school pleasant for everyone. Despite the focus of the question on good leaders, a profile of negative leaders was found. It is possible that this profile would have contained even more children if the question had not contained the word “good”. Thus, further research might also investigate whether differently formulated peer nomination questions on leadership distinguish between positive and negative leaders.

Further research might test other characteristics that distinguish leaders from non-leaders, and positive from negative leaders. For example, big five factors (e.g., extraversion) are relevant in adult leadership behaviors (Tackett et al., 2022). It might also be examined whether positive leaders contribute to a better and safer social atmosphere in the classroom, or whether an anti-bullying intervention works better in classrooms with positive rather than negative leaders. At the same time, it is relevant to investigate whether negative leaders can be turned into positive leaders (Ellis et al., 2016). The findings suggest that the relatively small differences in individual characteristics between positive and negative leaders may enable negative leaders to make the transition to positive leaders when their environment requires them to do so.

## Conclusion

Leaders are important as they give orders to classmates, make decisions in groups, and have power over others. The current study contributes to the literature through the discovery of three types of leaders: positive leaders, negative leaders, and non-popular leaders. Based on individual and interpersonal attributes, the findings affirm that leaders have higher self-esteem than non-leaders, and that interpersonal factors are the most effective in differentiating positive from negative leaders. The findings suggest that positive leaders show the ability to establish and sustain favorable relationships with others, whereas negative leaders lead others to accomplish their own goals by using aggression effectively. These findings have practical implications for educational professionals who wish to foster prosocial classroom norms; it may help teachers to realize that some popular children are negative leaders and may not contribute to the maintenance of positive classroom norms. When teachers wish to involve children in fostering a positive classroom atmosphere, they have to know not only which children are perceived as leaders, but also whether their behavior is positive or negative.

## Supplementary Information

Supplementary Information
