# Supplementary material for: Positive and Negative Leadership in Late Childhood: Similarities in Individual but Differences in Interpersonal Characteristics
Source: J Youth Adolesc. 2023 Jun 12;52(8):1620–31. doi: 10.1007/s10964-023-01798-3 (PMC10275811; doi:10.1007/s10964-023-01798-3)
Supplement: Supplementary file 1 — Supplementary Information [file 10964_2023_1798_MOESM1_ESM.docx]

**Appendix 1: Supplementary Latent Profile Analyses at T1**

This study replicated the latent profile analysis using the baseline data of KiVa NL (T1). Table S1 presents the fit statistics for the two- to eight-profile solutions using these data. The baseline sample consisted of 9,350 children (49.7% boys); there were no missing data. Again, a seven-profile solution was selected. The seven-profile solution had lower LL, AIC, BIC, and SSA-BIC than the two- to six-profile solutions. In addition, the seven-profile solution had reasonable sample sizes for each profile (*n* = 224 or more per profile), an acceptable entropy value of .842, and an acceptable LMR value lower than .05. More than 90% of children were classified accurately, and post hoc tests showed significant differences in most comparisons. The eight-profile solution was rejected because it failed in the LMR value.

The seven profiles were labeled according to the estimated z-standardized mean indicator variables (see Table S2). The largest profile was labeled as modal (*N*=5370, 44.0%): these children were characterized as having below-average levels of leadership, popularity, defending, and bullying.

The second and third profiles comprised positive and negative leaders; both were high on leadership and popularity, but they differed in defending and bullying. *Positive leaders* (*N*=366, 57.7% boys) were characterized by the highest level of defending and the lowest level of bullying. *Negative leaders* (*N*=356, 80.9% boys) showed medium levels of defending and high levels of bullying. Again, a profile of non-popular leaders was found. *Non-popular leaders* (*N*=881, 40.6% boys) had a high level of defending, above-average leadership, and relatively low levels of popularity and bullying.

A profile of *other popular children* (*N*=1229, 50.6% boys) was found as well. They had high levels of popularity, moderate levels of leadership and defending, and low levels of bullying. The final profiles consisted, again, of *pure bullies* and *extreme bullies.* The *pure bullies* (*N=*924, 68.6% boys) and *extreme bullies* (*N*=224, 77.2% boys) scored high on bullying, and (below) average on leadership, popularity, and defending.

**Table S1**

*Fit Statistics for Profile Structures at T1*

|  | LL | FP | AIC | BIC | SSA-BIC | LMR (*p*) | BLRT (*p*) | Entropy |
| --- | --- | --- | --- | --- | --- | --- | --- | --- |
| 2 | -49185.88 | 13 | 98397.75 | 98490.61 | 98449.30 | <.0001 | <.0001 | .882 |
| 3 | -48076.98 | 18 | 96189.96 | 96318.54 | 96261.34 | <.0001 | <.0001 | .877 |
| 4 | -47045.44 | 23 | 94136.88 | 94301.17 | 94228.08 | <.0001 | <.0001 | .898 |
| 5 | -46495.82 | 28 | 93047.65 | 93247.65 | 93158.68 | <.0001 | <.0001 | .888 |
| 6 | -46166.03 | 33 | 92398.06 | 92633.79 | 92528.93 | <.0001 | <.0001 | .842 |
| **7** | **-45913.35** | **38** | **91902.69** | **92174.13** | **92053.37** | **.0346** | **<.0001** | **.842** |
| 8 | -45451.39 | 43 | 90988.77 | 91295.93 | 91159.28 | .0688 | <.0001 | .848 |

The selected solution is in bold

*LL* log-likelihood, *FP* Number of free parameters, *AIC* Akaike information criterion, *BIC* Bayesian information criterion, *SSA-BIC* sample-size-adjusted BIC, *LMRT* Lo, Mendell, and Rubin (2001) likelihood ratio test, *BLRT* bootstrap likelihood ratio tests.

**Table S2**

*Descriptive Information per Latent Profile at T1*

| Type | Number | % of sample | % of boys | Leadership | Popularity | Defending | Bullying |
| --- | --- | --- | --- | --- | --- | --- | --- |
| 1. Modal | 5370 | 57.4 | 44.0 | -0.424 | -0.527 | -0.216 | -0.407 |
| 2. Positive Leaders | 366 | 3.9 | 57.7 | 1.908 | 2.331 | 0.968 | -0.720 |
| 3. Negative Leaders | 356 | 3.8 | 80.9 | 0.949 | 2.015 | 0.191 | 2.295 |
| 4. Non-popular leaders | 881 | 9.4 | 40.6 | 1.097 | 0.022 | 0.657 | -0.460 |
| 5. Other Popular Children | 1229 | 13.1 | 50.6 | 0.367 | 1.143 | 0.389 | -0.106 |
| 6. Bullies | 924 | 9.9 | 68.6 | -0.414 | -0.313 | -0.391 | 1.321 |
| 7. Extreme Bullies | 224 | 2.4 | 77.2 | -0.180 | 0.060 | -0.470 | 2.920 |

All indicators were *z*-standardized per classroom.
